# Do opposite ends of same factors underlie life satisfaction vs. depressive symptoms among older people?

**DOI:** 10.1007/s40520-020-01765-z

**Published:** 2021-01-27

**Authors:** Katja Pynnönen, Katja Kokko, Milla Saajanaho, Timo Törmäkangas, Erja Portegijs, Taina Rantanen

**Affiliations:** Faculty of Sport and Health Sciences, Gerontology Research Center, University of Jyväskylä, Jyväskylä, Finland

**Keywords:** Mental well-being, Emotional well-being, Life resources, Aged people

## Abstract

**Background:**

Although depressive symptoms are more common among older than younger age groups, life satisfaction tends to remain stable over the life course, possibly because the underlying factors or processes differ.

**Aim:**

To study whether the factors that increase the likelihood of high life satisfaction also decrease the likelihood of depressive symptoms among older people.

**Methods:**

The data were a population-based probability sample drawn from community-dwelling people aged 75, 80, and 85 years (*n* = 1021). Participants’ life satisfaction was measured with the Satisfaction with Life Scale and depressive symptoms with the Centre for Epidemiologic Studies Depression Scale (CES-D). Physical performance, perceived financial situation, executive functions, loneliness, self-acceptance, and having interests in one’s life were studied as explanatory variables. The data were analyzed using cross-sectional bivariate linear modeling.

**Results:**

Better physical performance, not perceiving loneliness, having special interests in one’s life, and higher self-acceptance were associated with higher life satisfaction and fewer depressive symptoms. Better financial situation was related only to life satisfaction. Executive functions were not associated with either of the outcomes.

**Discussion:**

The opposite ends of the same factors underlie positive and negative dimensions of mental well-being.

**Conclusion:**

Further studies are warranted to better understand how people maintain life satisfaction with aging when many resources may diminish and depressive symptoms become more prevalent.

**Supplementary Information:**

The online version contains supplementary material available at 10.1007/s40520-020-01765-z.

## Introduction

Maintaining mental well-being and mental health is an essential component of aging well. Mental well-being comprises high levels of emotional, psychological, and social well-being, and not merely the absence of mental illness [1, 2]. Mental well-being may become threatened by processes that often coincide with aging, such as loss of essential life resources [3–7]. According to the resource-based dynamic perspective, resources can be defined as an individual’s total capability to fulfill his or her centrally valued needs and include a multitude of factors [8]. In addition to instrumental value, resources also have symbolic value helping people to define who they are [9] and contributing to peoples’ life satisfaction judgements [10]. People usually aim at retaining, protecting, and building resources, and the loss of valued resources may induce psychological stress and trigger mental ill health in already vulnerable individuals [9, 11]. In this study, we focus on the question whether the same life resources when present increase the likelihood of high mental well-being, i.e., life satisfaction, and when absent increase the likelihood of low mental well-being, i.e., depressive symptoms among older people.

Life satisfaction is a rather stable experience that includes a cognitive judgment on how satisfied one is with one’s life as a whole [12], whereas depressive symptoms are typically episodic normal adjustment reactions to negative life events [13] which over time can hinder living a normal life [14]. Earlier studies have shown that depressive symptoms become more prevalent with aging [15, 16]. Life satisfaction, instead, may be more stable. Some studies show that life satisfaction remains rather constant across the life span up to very old age [17, 18], while others suggest that it increases up to age 65–70 years and then declines [19].

There is some earlier evidence suggesting that absence or presence of similar life resources correlate with positive and negative aspects of mental well-being, respectively. However, the evidence mostly come from studies addressing sole one or the other aspect of mental well-being. Physical and financial resources, such as disability, mobility limitations, or perceived poor financial situation, are associated with lower life satisfaction [4, 5, 20, 21] and an increased risk for depressive symptoms [5, 6, 22, 23]. Cognitive decline increases the risk for depressive symptoms [6], but its association with life satisfaction remains unknown. Loneliness, an indicator of lack of social resources, is related to lower life satisfaction [3, 24, 25] and increased risk for depressive symptoms [7, 26, 27]. Self-acceptance, an indicator of psychological resources, correlates with life satisfaction, depression, and morale among people of different ages [28, 29], but multivariate analyses adjusting for potential confounders have not been reported. The associations of having special interests in one’s life with life satisfaction and depressive symptoms have not been previously studied, even though loss of interest in previously enjoyable activities is a symptom of depression [30].

In summary, satisfaction with life and depressive symptoms are distinct dimensions of mental well-being and appear to show different age-related patterns. Although only a few studies have simultaneously addressed both seem to be related to individuals’ physical, financial, social, and psychological resources. Thus, the purpose of this study was to explore whether the physical (physical performance), financial (perceived financial situation), cognitive (executive functions), social (not perceiving loneliness) and psychological (self-acceptance and having interests in one’s life) resources are associated positively with life satisfaction and negatively with depressive symptoms among 75-, 80-, and 85-year-old people.

## Materials and methods

### Study design and participants

This population-based cross-sectional study is part of the Active aging—resilience and external support as modifiers of the disablement outcome (AGNES) cohort study. Participants comprised a population-based probability sample of community-dwelling people (*n* = 1021) aged 75, 80, and 85 years living in the city of Jyväskylä, Central Finland. The data of the present study were collected in face-to-face home interviews, by postal questionnaires, and by measurements conducted at the research center between September 2017 and December 2018. Exclusion criteria were unwillingness to participate and inability to communicate. The study design and data collection have been described in detail earlier [31, 32].

### Measures

*Life satisfaction* was measured using the Satisfaction with Life Scale (SWLS) which assesses a person’s overall judgement of his or her life [12]. The scale has five items, including ‘I am satisfied with my life’ and ‘In most ways my life is close to my ideal’, each rated on a 7-point Likert scale from 1 = strongly disagree to 7 = strongly agree. In sum scores (range 5–35), higher scores indicate higher satisfaction. Cronbach’s alpha was 0.89.

*Depressive symptoms* were assessed with the 20-item Centre for Epidemiologic studies Depression Scale (CES-D) [33], with items on four dimensions: depressed affect (e.g., feeling depressed, fearful), somatic symptoms (e.g., poor appetite, trouble concentrating), positive affect (e.g., feeling as good as others, enjoying life), and interpersonal problems (e.g., people were unfriendly). Participants are asked to rate the frequency of each depressive symptom during the previous week on a 4-point scale ranging from 0 = rarely or never to 3 = almost all the time. In sum scores (range 0–60), higher scores indicate more depressive symptoms. A sum score was calculated for participants with at most one missing item. Cronbach’s alpha was 0.88.

Physical resources (*lower extremity physical performance*) were measured with the Short Physical Performance Battery (SPPB), including tests for standing balance, normal gait speed over 3 m, and chair rise time [31, 34]. Each test is scored from 0 to 4, and test scores are summed (range 0–12), with higher scores indicating better physical performance. Cronbach’s alpha was 0.72. Cognitive resources (e*xecutive functions*) were assessed with the Trail Making Tests parts A and B [35]. Executive functions are the high-level cognitive processes that control and organize other mental processes and enable flexible behavior [36]. The Trail Making Tests evaluate information and visual search, scanning, processing speed, mental flexibility, and executive functioning. In part A, the participant is required to connect as quickly as possible and in ascending order a series of randomly dispersed circles containing numbers. In part B, the participant is required to connect as quickly as possible and in alternating sequential order a series of randomly dispersed circles containing numbers and letters. The maximum time taken to complete each part of the test was 300 s. The difference in the time taken to accomplish Parts B and A was calculated, with smaller time differences indicating better performance. Financial resources (*perceived financial situation*) were measured with a single question with the response options 1 = poor, 2 = moderate, 3 = good, or 4 = very good. Social resources were measured with a single question “How often do you *feel lonely*?” [37]. Responses were coded as 1 = very rarely/never, 2 = rarely, or 3 = often/almost always. Psychological resources were evaluated with a single item from the Scales of Psychological Well-Being *self-acceptance* subscale assessing self-confidence and positive attitude to oneself [38]. On the six-point rating scale, a higher score indicates stronger agreement with statement. Psychological resources were also evaluated according to the extent to which the participant agreed or disagreed with the statement “I have special interests in my life” [31]. The responses were coded 1 = strongly or quite strongly disagree, 2 = do not agree/disagree, 3 = quite strongly agree, and 4 = strongly agree. Self-reported physician-diagnosed chronic conditions were ascertained with a help of a list of common chronic conditions prompted by ten disease categories. A *morbidity index* was then calculated as a sum of individual chronic conditions reported. Information on *age* and *sex* (0 = men, 1 = women) was drawn from the population register.

### Statistical analysis

The outcome variable distributions were skewed, and hence the CES-D score was log-transformed and the life satisfaction score raised to the power of 1.8. Life satisfaction and depressive symptoms were analyzed as correlated outcomes in a bivariate linear model in Mplus 5.2 [39]. Physical performance, perceived financial situation, executive functions, feelings of loneliness, self-acceptance, and having interests in one’s life were used as predictor variables for the two outcomes. The parameter estimates were tested with the single-parameter Wald test, thus increasing the possibility for false-positive findings. To guard against this, we used the method introduced by Šidák [40] to control for the familywise error rate and to compute *p* values corrected for multiple testing. Statistical significance was set at *p* < 0.05. The likelihood ratio test was used to test for statistically significant group differences in the regression coefficients across the various age groups or between men and women. The model was adjusted for morbidity index. We assumed the missing responses were generated by the missing-at-random mechanism and used the maximum likelihood estimator to account for missingness. The maximum number of missing observations for a variable pair was 288 (28.2%).

## Results

Descriptive characteristics are shown in Table 1. Of the participants, 45% were aged 75, 33% 80 and 22% 85 years, and 57% were women. The mean number of depressive symptoms was 8.6 (standard deviation SD 7.1), and the mean score for satisfaction with life 26.6 (SD 5.4). The mean score for physical performance was 9.9 (SD 2.4), the average number of morbidity index was 3.41 (SD 2.0), and the mean difference in the time taken to accomplish Parts B and A in the test of executive functions was 83.8 s (SD 43.4). Approximately 60% of the participants rated their financial situation as good or very good, 38% as moderate, and 2% as poor. Half of the participants reported having feelings of loneliness never/very rarely, 37% rarely, and 7% often/almost always. Nine out of ten participants agreed at least quite strongly with the statement about having special interests in their life, and approximately 60% with the statement about having self-confidence and positive attitude to their self (score 5 or 6).

**Table 1:** Descriptive data of the AGNES-cohort participants (*n* = 1021)

	Mean	Standard deviation
Life satisfaction (range 5–35)	26.57	5.38
Depressive symptoms (range 0–60)	8.64	7.09
Physical performance (range 0–12)	9.87	2.39
Executive functions, TMTΔ (s)	83.78	43.40
Morbidity index (number)	3.41	2.04

	*n*	%
Age		
75 years	458	44.9
80 years	336	32.9
85 years	227	22.2
Women	585	57.3
Perceived financial situation		
Poor	17	1.7
Moderate	381	37.9
Good	505	50.3
Very good	101	10.1
Loneliness		
Never/very rarely	561	56.1
Rarely	372	37.2
Often/almost always	67	6.7
Self-acceptance		
1 (strongly disagree)	4	0.4
2	26	2.7
3	88	9.1
4	281	29.1
5	376	38.9
6 (strongly agree)	192	19.9
Having interests in one’s life		
Strongly or quite strongly disagree	56	5.5
Not agree or disagree	49	4.8
Quite strongly agree	494	48.6
Strongly agree	417	41.0

Higher life satisfaction correlated with fewer depressive symptoms (*r* = − 0.485, *p* value < 0.001) (Supplementary Table 1). The results of the bivariate linear model indicated that better physical performance, less often perceiving oneself as lonely, having special interests in one’s life, and having higher self-acceptance were positively associated with higher life satisfaction and negatively with a higher number of depressive symptoms (Table 2). Participants who reported perceiving loneliness often/almost always or rarely were less satisfied with their life and had a higher number of depressive symptoms than those who reported experiencing loneliness very rarely/never. When compared with those who strongly agreed with the statement about having special interests in life, those who did not agree/disagree or who strongly/quite strongly disagreed with the statement reported lower life satisfaction. On the other hand, those who quite strongly agreed or did not agree/disagree with the statement differed in their level of depressive symptoms from those who strongly agreed with it. In the self-acceptance ratings, all participants with the exception of those who strongly disagreed with the statement about self-acceptance had lower life satisfaction and a higher number of depressive symptoms compared to those who strongly agreed with it (i.e., reported high self-acceptance). Executive functions were not associated with either of the outcomes. Better perceived financial situation was associated solely with higher life satisfaction: those who rated their financial situation as moderate or poor reported lower life satisfaction than those who rated it as very good. Adjusting the model for morbidity index did not materially change the results.

**Table 2:** Standardized parameter estimates, standard errors, *p* values, and 95% confidence intervals for the bivariate linear model of the associations of various resources with life satisfaction and depressive symptoms in the AGNES-cohort participants

Life satisfaction as an outcome				Sidak-adjusted *p* value	Nominal 95% confidence intervals	
Parameter	Estimate	SE	*p* value		Lower	Upper
Physical performance	**0.139**	**0.030**	**< 0.001**	**< 0.001**	**0.081**	**0.197**
Perceived financial situation						
Good vs. very good	− 0.108	0.046	0.019	0.499	− 0.197	− 0.018
Moderate vs. very good	**− 0.262**	**0.046**	**< 0.001**	**< 0.001**	**− 0.352**	**− 0.171**
Poor vs. very good	**− 0.111**	**0.030**	**< 0.001**	**0.007**	**− 0.170**	**− 0.053**
Executive functioning	− 0.001	0.033	0.976	1.000	− 0.066	0.064
Loneliness						
Rarely vs. very rarely/never	**− 0.170**	**0.028**	**< 0.001**	**< 0.001**	**− 0.225**	**− 0.116**
Often/almost always vs. very rarely/never	**− 0.245**	**0.028**	**< 0.001**	**< 0.001**	**− 0.301**	**− 0.190**
Self-acceptance						
5 vs. 6	**− 0.176**	**0.037**	**< 0.001**	**< 0.001**	**− 0.249**	**− 0.104**
4 vs. 6	**− 0.221**	**0.037**	**< 0.001**	**< 0.001**	**− 0.293**	**− 0.149**
3 vs. 6	**− 0.216**	**0.032**	**< 0.001**	**< 0.001**	**− 0.279**	**− 0.153**
2 vs. 6	**− 0.123**	**0.029**	**< 0.001**	**0.001**	**− 0.180**	**− 0.065**
1 vs. 6	− 0.059	0.027	0.032	0.697	− 0.113	− 0.005
Having interests in one’s life						
Quite strongly agree vs. strongly agree	− 0.059	0.029	0.042	0.798	− 0.116	− 0.002
Not agree or disagree vs. strongly agree	**− 0.097**	**0.028**	**0.001**	**0.020**	**− 0.153**	**− 0.042**
Strongly/quite strongly disagree vs. strongly agree	**− 0.159**	**0.030**	**< 0.001**	**< 0.001**	**− 0.217**	**− 0.101**
Age						
80 years vs. 75 years	0.011	0.029	0.698	1.000	− 0.045	0.067
85 years vs. 75 years	0.078	0.032	0.015	0.425	0.015	0.140
Sex	− 0.024	0.027	0.383	1.000	− 0.077	0.030

Depressive symptoms as an outcome						
Physical performance	**− 0.185**	**0.030**	**< 0.001**	**< 0.001**	**− 0.243**	**− 0.127**
Perceived financial situation						
Good vs. very good	0.070	0.046	0.129	0.994	− 0.020	0.160
Moderate vs. very good	0.137	0.047	0.003	0.116	0.046	0.228
Poor vs. very good	0.053	0.030	0.072	0.938	− 0.005	0.111
Executive functioning	− 0.004	0.032	0.897	1.000	− 0.066	0.058
Loneliness						
Rarely vs. very rarely/never	**0.174**	**0.028**	**< 0.001**	**< 0.001**	**0.119**	**0.229**
Often/almost always vs. very rarely/never	**0.214**	**0.029**	**< 0.001**	**< 0.001**	**0.158**	**0.271**
Self-acceptance						
5 vs. 6	**0.177**	**0.037**	**< 0.001**	**< 0.001**	**0.105**	**0.249**
4 vs. 6	**0.262**	**0.037**	**< 0.001**	**< 0.001**	**0.190**	**0.333**
3 vs. 6	**0.236**	**0.032**	**< 0.001**	**< 0.001**	**0.173**	**0.298**
2 vs. 6	**0.171**	**0.028**	**< 0.001**	**< 0.001**	**0.116**	**0.227**
1 vs. 6	0.075	0.028	0.006	0.211	0.021	0.130
Having interests in one’s life						
Quite strongly agree vs. strongly agree	**0.102**	**0.029**	**< 0.001**	**0.017**	**0.045**	**0.159**
Not agree or disagree vs. strongly agree	**0.103**	**0.028**	**< 0.001**	**0.009**	**0.048**	**0.159**
Strongly/quite strongly disagree vs. strongly agree	0.091	0.031	0.003	0.113	0.030	0.151
Age						
80 years vs. 75 years	0.001	0.029	0.967	1.000	− 0.055	0.058
85 years vs. 75 years	0.000	0.032	0.992	1.000	− 0.062	0.062
Sex	0.035	0.027	0.203	1.000	− 0.019	0.088

Statistically significant values are in bold

Although the 85-year-old participants had more depressive symptoms than the 75-year-olds (tested with one-way ANOVA; *F* = 5.487, *p* value = 0.004), age was not associated with depressive symptoms in the final bivariate linear model. The three age groups did not differ in life satisfaction (*F* = 1.452, *p* value = 0.235). Similarly, sex was not related to either outcome in the final model, although men had slightly fewer depressive symptoms (tested with independent samples *t* test; *t* = − 2.267, *p* value = 0.024) and higher life satisfaction (*t* = 2.921, *p* value = 0.004) than women. The likelihood ratio test for the null hypothesis of equal regression coefficients across the age and gender groups was not statistically significant ($\chi_{df}^{2} = 10$, *p* = 0.060), indicating no statistically significant group differences in the regression coefficients across these groups.

After including all the predictors in the model, the residual covariance between life satisfaction and depressive symptoms was statistically significant (standardized *β* = − 0.27; Sidak-adjusted *p* value < 0.001). The predictor variables explained 32% of the variation in life satisfaction and 30% of the variation in depressive symptoms.

## Discussion

The main result was that the same resources that were positively related to life satisfaction were negatively related to depressive symptoms. Thus, both the negative and positive aspects of mental well-being are related to the absence or presence of the resources needed to fulfill the requirements for leading a full life, respectively. The exception was that a better perceived financial situation predicted only higher life satisfaction. A novel feature of the current study is that we examined the simultaneous associations of several life resources with positive and negative dimensions of mental well-being among people in the same study, while earlier studies have mostly targeted only one or the other outcome or fewer resources.

Our results are mostly in line with earlier studies. The importance of physical resources for life satisfaction [4, 5, 21] and depressive symptoms [6, 23] has been reported earlier. Similarly, the present association between financial resources and life satisfaction was in line with earlier findings [4, 5, 41]. In contrast, we found no association between financial situation and depressive symptoms. Physical and financial resources are important in fulfilling people’s very basic needs and may also facilitate well-being by enabling individuals to pursue and attain goals important to them [42, 43]. Thus, these resources have instrumental value for older people [45]. Physical performance may also describe impairment in health [34], a known correlate for depressive symptoms among older people [6]. In contrast to earlier reports [6, 46] we found no association between cognitive functioning and depressive symptoms. This may be explained by differences in the methodologies used. Executive functions, supported by the prefrontal cortex [36] may be the first to decline with increasing age [46], while in other studies tests capturing more manifest cognitive declines may have been used. Another explanation may be differences in participant populations.

Lack of social resources, i.e., perceived loneliness, was associated with more severe depressive symptoms, while not perceiving loneliness coincided with higher life satisfaction [7, 24–26]. Experiencing connectedness to others and having good social relationships with family, friends, and neighbors contribute to good quality of life [44]. The absence of social resources, i.e., not having anyone to count on for help when needed or not feeling respected are detrimental to emotional well-being [41]. Feelings of loneliness may be associated with unmet expectations and dissatisfaction with contacts with friends and children [47].

Our bivariate results strengthened the earlier knowledge of an association of self-acceptance with life satisfaction and depressive symptoms [28, 29] among older people. This is logical, because self-acceptance is a central feature of mental health and positive psychological functioning [29, 48] including acceptance of oneself and of one’s past life, a positive attitude towards oneself, and awareness and acceptance of personal strengths and limitations. Having special interests in one’s life indicates involvement in life situations, being inspired, and having the will to learn and grow personally. In an earlier study, learning new things and being able to choose how to spend one’s time were positively related to life satisfaction [41]. A high level of psychological resources, in the current study self-acceptance and having special interests, may have symbolic value in aiding self-definition [9] while a low level of these resources may render an individual’s mental well-being more vulnerable to impairment.

In the age-group comparisons, the 85-year olds, the oldest age group, reported more depressive symptoms than the 75-year olds, the youngest age group. However, in the model including all the predictor variable age was not associated with depressive symptoms. This might indicate that having more depressive symptoms in very old age is not due to increased age, but rather to lower levels of physical, social, and financial resources. Another age-related observation was that the mean-level of life satisfaction was similar across the three age groups. This outcome has been called ‘the satisfaction paradox’ [43]. The ability to cope with and adapt to adversities and changes in one’s life helps people to maintain an adequate level of emotional well-being. In addition, it is possible that older people adjust their reference point to what they consider is realistic in their current circumstances, a practice which may eventually fuel adaptation even in the face of losses in resources [43, 49].

This study has both strengths and limitations. A limitation that may restrict the generalizability of the results was that the participants were on average somewhat healthier and had better functioning than those who declined to participate [32]. This may lead to slight under-estimation of the strength of the associations. The data were collected by means of a computer-assisted face-to-face personal interview conducted in the participants’ homes, thereby facilitating the participation of those with poorer health and function and assuring the ability to participate also for those with poorer health. A second limitation was the use of cross-sectional data, which does not allow conclusions to be drawn on causality between the variables. A third limitation is that the factors predicting life satisfaction and depressive symptoms may differ in different cultures. For example, in Western countries, the concept of mental well-being may be more dependent on individual and personal qualities than is the case, for instance, in Asian countries [2]. There are other factors known to be connected with life satisfaction besides those addressed in our study. For example, personality traits are strong predictors of life satisfaction [24, 50]. A strength was the use of a large population-based sample with sufficient numbers of men and women participants in the three age cohorts. The present results both strengthen and broaden earlier knowledge on the explanatory factors of life satisfaction and depressive symptoms in older people. To better understand how people maintain life satisfaction when aging, when resources often diminish and depressive symptoms become more prevalent, calls for further research.

## Conclusion

Having positive life resources correlates with positive dimension of mental well-being, while the absence of the same resources correlates with the negative dimension of mental well-being. The results suggest that by reinforcing older people’s ability to maintain their physical, financial, social, and psychological resources may help them to maintain their mental well-being.

## Supplementary Information

Below is the link to the electronic supplementary material.

Supplementary file1 (DOCX 17 KB)

## Data Availability

The data used in this study are available by request from the authors.
