# Supplementary material for: Do opposite ends of same factors underlie life satisfaction vs. depressive symptoms among older people?
Source: Aging Clin Exp Res. 2021 Jan 27;33(9):2557–64. doi: 10.1007/s40520-020-01765-z (PMC8429157; doi:10.1007/s40520-020-01765-z)
Supplement: Supplementary file 1 — Supplementary file1 (DOCX 17 KB) [file 40520_2020_1765_MOESM1_ESM.docx]

**Aging Clinical and Experimental Research**

**Do opposite ends of same factors underlie life satisfaction vs. depressive symptoms among older people?**

Katja Pynnönen^1^, Katja Kokko^1^, Milla Saajanaho^1^, Timo Törmäkangas^1^, Erja Portegijs^1^, Taina Rantanen^1^

^1^Faculty of Sport and Health Sciences, Gerontology Research Center, University of Jyväskylä, Jyväskylä, Finland

Corresponding author: Katja Pynnönen, katja.pynnonen@jyu.fi

**Supplementary table 1.** Mean and standard deviation of the study variables, and Pearson’s correlations between resource factors, life satisfaction, and depressive symptoms in the AGNES-cohort participants

|  | **Mean** | **Standard deviation** | **Correlation coefficients** | |
| --- | --- | --- | --- | --- |
|  |  |  | **Life satisfaction** | **Depressive symptoms** |
| **Life satisfaction** | 26.57 | 5.38 | 1 |  |
| **Depressive symptoms** | 8.64 | 7.09 | -.485 | 1 |
| **Physical performance** | 9.87 | 2.39 | .234 | -.281 |
| **Morbidity index** | 3.41 | 2.04 | -.228 | .227 |
| **Perceived financial situation dummy1^1^** | 0.50 | 0.50 | .148 | -.095 |
| **Perceived financial situation dummy2^2^** | 0.38 | 0.49 | -.235 | .167 |
| **Perceived financial situation dummy3^3^** | 0.02 | 0.13 | -.100 | .056 |
| **Executive functioning** | 83.78 | 43.40 | -.076 | .079 |
| **Feelings of loneliness dummy1^4^** | 0.37 | 0.48 | -.184 | .200 |
| **Feelings of loneliness dummy2^5^** | 0.07 | 0.25 | -.293 | .267 |
| **Self-acceptance dummy1^6^** | 0.39 | 0.49 | .078 | -.105 |
| **Self-acceptance dummy2^7^** | 0.29 | 0.45 | -.118 | .147 |
| **Self-acceptance dummy3^8^** | 0.09 | 0.29 | -.207 | .199 |
| **Self-acceptance dummy4^9^** | 0.03 | 0.16 | -.107 | .147 |
| **Self-acceptance dummy5^10^** | 0.00 | 0.06 | -.094 | .096 |
| **Having interests in life dummy1^11^** | 0.49 | 0.50 | -.058 | .115 |
| **Having interests in life dummy2^12^** | 0.05 | 0.21 | -.119 | .121 |
| **Having interests in life dummy3^13^** | 0.06 | 0.23 | -.220 | .161 |
| **Age 80 vs. 75-years** | 0.33 | 0.47 | -.013 | -.001 |
| **Age 85 vs. 75-years** | 0.22 | 0.42 | -.043 | .099 |
| **Sex** | 0.57 | 0.50 | -.093 | .095 |

Note. ^1^: 1=good, 0=poor, moderate, or very good; ^2^: 1=moderate, 0=poor, good, or very good; ^3^: 1=poor, 0=moderate, good, or very good; ^4^: 1=rarely, 0=often/almost always or never/very rarely; ^5^: 1=often/almost always, 0=rarely or never/very rarely; ^6^: 1=5, 0=1, 2, 3, 4, 6; ^7^: 1=4, 0=1, 2, 3, 5, 6; ^8^: 1=3, 0=1, 2, 4, 5, 6; ^9^: 1=2, 0 = 1, 3, 4, 5, 6; ^10^: 1=1, 0 = 2, 3, 4, 5, 6; ^11^: 1=quite strongly agree, 0=strongly/quite strongly disagree, not agree/disagree, or strongly agree; ^12^: 1=not agree/disagree, 0 =strongly/quite strongly disagree, quite strongly agree, or strongly agree; ^13^: 1=strongly/quite strongly disagree, 0=not agree/disagree, quite strongly agree, or strongly agree.
